# Coexistence of metabolic-associated fatty liver disease and autoimmune or toxic liver disease

**DOI:** 10.1097/MEG.0000000000002785

**Published:** 2024-04-26

**Authors:** Oscar Danielsson, Tiina Vesterinen, Johanna Arola, Fredrik Åberg, Markku J. Nissinen

**Affiliations:** a Clinic of Gastroenterology, Abdominal Center, Helsinki University Hospital and University of Helsinki; b Doctoral Programme in Clinical Research, University of Helsinki; c HUS Diagnostic Center, HUSLAB, Helsinki University Hospital and University of Helsinki; d Department of Pathology, Faculty of Medicine, University of Helsinki; e Abdominal Center, Transplantation and Liver Surgery, Helsinki University Hospital and University of Helsinki, Helsinki, Finland

**Keywords:** autoimmune hepatitis, primary biliary cholangitis, primary sclerosing cholangitis, toxic liver injury

## Abstract

Fatty liver disease (FLD) affects approximately 25% of global adult population. Metabolic-associated fatty liver disease (MAFLD) is a term used to emphasize components of metabolic syndrome in FLD. MAFLD does not exclude coexistence of other liver disease, but impact of coexisting MAFLD is unclear. We investigated prevalence and characteristics of MAFLD in patients with biopsy-proven autoimmune hepatitis (AIH), primary biliary cholangitis (PBC), primary sclerosing cholangitis (PSC), or toxic liver disease. Liver histopathology and clinical data from Helsinki University Hospital district (1.7 million inhabitants) between 2009 and 2019 were collected from patients with AIH, PBC, PSC, or toxic liver disease at the time of diagnosis. MAFLD was diagnosed as macrovesicular steatosis ≥5% together with obesity, type-2 diabetes, or signs of metabolic dysregulation. Of 648 patients included, steatosis was observed in 15.6% (*n* = 101), of which 94.1% (*n* = 95) was due to MAFLD. Prevalence of coexisting MAFLD in the four liver diseases varied between 12.4 and 18.2% (*P* = 0.483). Fibrosis was more severe in MAFLD among patients with toxic liver disease (*P* = 0.01). Histopathological characteristics otherwise showed similar distribution among MAFLD and non-FLD controls. Alcohol consumption was higher in MAFLD group among patients with AIH or PBC (*P* < 0.05 for both). In AIH, smoking was more common in patients with coexisting MAFLD (*P* = 0.034). Prevalence of coexisting MAFLD in other primary liver diseases is lower than reported in general population. Histopathology of MAFLD patients did not clearly differ from non-FLD ones. Alcohol and smoking were associated with MAFLD in AIH.

## Introduction

Fatty liver disease (FLD) is the most common chronic liver disease and affects approximately 25% of the global adult population and presents complications including nonalcoholic steatohepatitis (NASH), fibrosis, cirrhosis, end-stage liver disease, hepatocellular carcinoma and liver-related death [1,2]. FLD is associated with sedentary lifestyle, obesity, type-2 diabetes (T2DM) and metabolic dysregulation [3]. The risk of disease progression of fatty liver into cirrhosis and its complications is substantially higher in the presence of NASH, which is defined as lobular inflammation and hepatocellular ballooning in the presence of steatosis [4,5].

Although liver biopsy is the gold standard for quantifying liver steatosis, this procedure has serious risks and is expensive. This has driven the development of indices and other noninvasive tools to assess the risk of steatosis and fibrosis. The diagnosis of FLD in clinical practice still relies on imaging examinations, principally liver ultrasound [6].

To emphasize the role of metabolic factors behind nonalcoholic fatty liver disease (NAFLD) and to exclude dichotomized division into NAFLD and alcohol-related liver disease, the term metabolic-associated fatty liver disease (MAFLD) has been suggested [7]. MAFLD is defined as FLD in combination with either overweight (BMI > 25 kg/m²), T2DM, or at least two signs of metabolic dysregulation [7]. FLD does not exclude the risk of other liver disease and vice versa. Although FLD may affect the clinical course of patients with any other liver disease, few studies have addressed this issue. Several components of metabolic syndrome are related to overall and liver-related mortality in persons with chronic liver disease [8].

In this study, we investigated clinical characteristics and liver biopsy reports in adult patients with an established diagnosis of autoimmune hepatitis (AIH), primary biliary cholangitis (PBC), primary sclerosing cholangitis (PSC), or toxic liver disease from a hospital district comprising 1.7 million adult inhabitants over a period of 10 years. The first aim of the study was to establish the prevalence of MAFLD in AIH, PBC, PSC and toxic liver disease. The second aim was to assess whether patients with coexisting MAFLD differ from non-FLD patients in terms of lifestyle factors (alcohol, smoking), liver-related blood tests, noninvasive tests (NIT), or liver histopathology at the time of diagnosis of the primary liver disease.

## Patients and methods

### Study sample

This cross-sectional retrospective study was based on liver biopsies taken at the Hospital district of Helsinki and Uusimaa (HUS) between 1 June 2009 and 31 May 2019 at the time of diagnosis of AIH, PBC, PSC or toxic liver disease. The procedures and study design were approved by the Institutional Review Board of HUS (license code HUS/115/2020).

The sample was gathered by searching the patient register (HUS Cressida) for patients with an entry for ICD-10 codes for AIH (K73.2, K75.4), PBC (K74.3), PSC (K83.0) or toxic liver disease (K71.0–K71.9) within the designated timeframe. The term toxic liver disease used here refers to liver injury induced by drug(s) or toxic agents, after the exclusion of other causative factors, for example, acute viral hepatitis [9]. Patients with an entry of any of these ICD-10 codes prior to 1 June 2009 were excluded as well as patients with an entry for liver cirrhosis (K70.3 or K74.6), liver transplantation (Z94.4) or liver cancer (C22.0–C22.9) within 6 months from initial diagnosis. Patients aged <16 years at the time of the first diagnosis entry were also excluded. This search yielded 3088 patients.

**Fig. 1.**
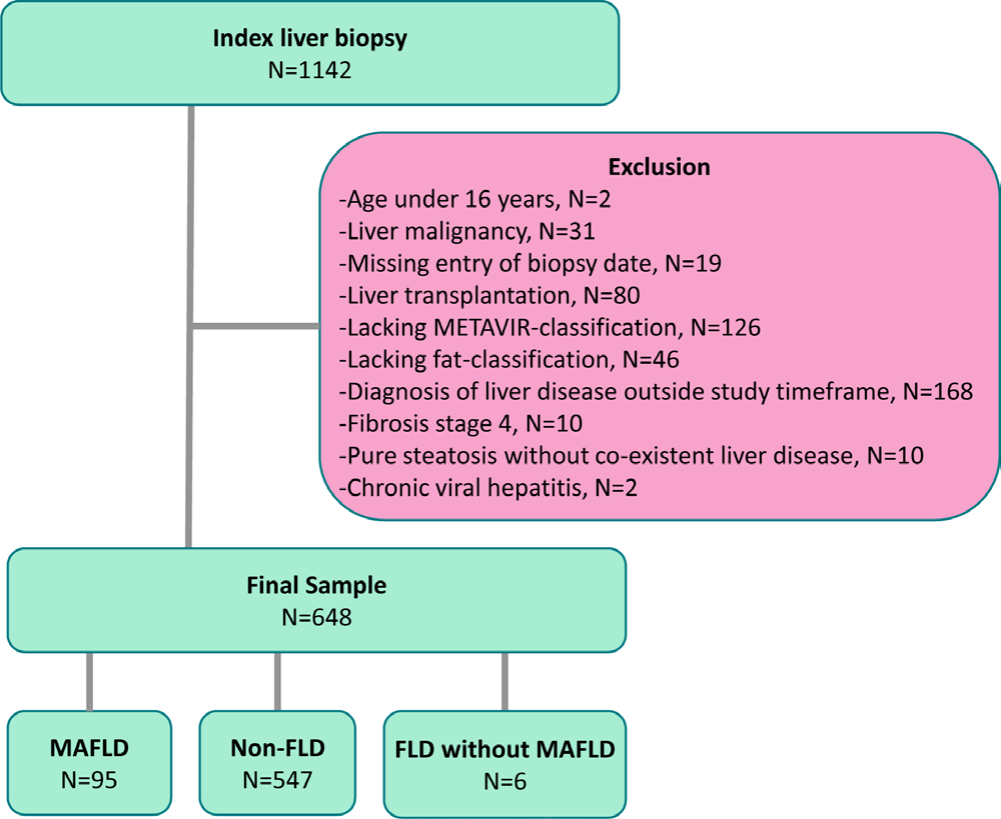
Study overview.

This list of the patients was then cross-checked with the liver biopsy register (QPati software, TietoEvry, HUS Diagnostic Center HUSLAB, Department of Pathology, Helsinki University Hospital) to identify patients with liver biopsy data (*n* = 1142). Liver histology was assessed as part of clinical routine using a structural report based on the METAVIR [10] classification. This included semiquantitative classification of inflammation (0–3), fibrosis (0–4), interphase activity (0–3), lobular activity (0–3), macro- and microvesicular steatosis (%), steatohepatitis (0–2) and cytokeratin 7 stain (0–3). After exclusions due to, for example, lacking data or liver transplantation, the final study sample consisted of 648 patients (Fig. 1).

Some AIH patients had entries for several diagnoses; if a patient had several entries for AIH and PBC or PSC, they were diagnosed as AIH-PBC overlap or AIH-PSC overlap, respectively, which was proven to be accurate after manual browsing of the register. For statistical analyses, overlap diseases were analyzed together with AIH.

### Health data

With assistance from HUS information technology management, we searched the patient records for laboratory test results, ICD-10 diagnosis entries, and data on weight, height and BMI. The search was limited to identify results from within 1 month before the first liver biopsy for the following blood tests: blood cell count, alanine aminotransferase (ALT), aspartate aminotransferase (AST), alkaline phosphatase (ALP), gamma-glutamyl transferase (γ-GT), ferritin, C-reactive protein (CRP), creatinine, albumin and thromboplastin time. Results ±3 months of first liver biopsy for the following blood tests were included: glycated hemoglobin (HbA1c), total cholesterol, high-density lipoprotein cholesterol (HDL-C), low-density lipoprotein cholesterol (LDL-C), and triglycerides. Of data on weight, height and BMI, results registered within a year of the first liver biopsy were included.

All diagnoses registered in medical records of the final study sample during the 10-year study period were listed. Comorbidity was recorded as ICD-10 registry entries for T2DM (E11.0–E11.9), type 1 diabetes (E10.0–E10.9), hypertension (I10) and dyslipidaemia (E78.5).

Fibrosis-4 index (FIB-4; age, ALT, AST and platelet count), APRI (ALT and platelet count), hepatic steatosis index (HSI [11], ALT, AST, BMI, sex and diabetes) and dynamic aspartate-to-alanine aminotransferase ratio (dAAR) [12] were calculated for patients with available data (Supplementary Table 1, Supplemental Digital Content 1, http://links.lww.com/EJGH/B28 for equations). In the FLD-patient group, FIB-4 index, APRI, HSI and dAAR were missing in 10, 10, 10 and 9 patients, respectively (Supplementary Table 2, Supplemental Digital Content 1, http://links.lww.com/EJGH/B28 for missing data).

Searches were completed manually by collecting all clinical details from the patient records for all FLD patients (*n* = 101) and from the age-, disease-, and sex-matched (*n* = 104) control group. In the MAFLD-patients and matched controls, the medications were manually registered from hospital records. Biopsies were taken as part of the diagnostic pathway before starting liver-specific medications, such as corticosteroids.

In Finland, alcohol consumption is systematically recorded in hospital records for each patient undergoing liver biopsy. Alcohol consumption was registered as abstinent, social drinker or heavy drinker according to registry entries on alcohol use. The term heavy drinker was defined as follows: (a) recorded mean regular high amount of weekly alcohol consumption (over 21 doses for men and over 14 doses for women, (b) frequent binge-drinking (over 5 drinks on occasion) behavior or (c) medical problems caused by alcohol drinking habits (e.g. hospitalizations or traumatic injuries due to alcohol consumption) [13,14]. Smoking status was also determined based on registry entries as nonsmoker or current or former smoker.

### Imaging data

By chart review, results from imaging studies (ultrasound, computed tomography or MRI) performed around the time of first liver biopsy were registered with regards to steatosis. Steatosis was registered if it was mentioned in the radiologist’s statement as steatosis or as increased echoing of the liver parenchyma (for ultrasound).

### Metabolic-associated fatty liver disease criteria

FLD was defined as ≥5% macrovesicular fat in the hepatocytes of the liver biopsy sample [15]. MAFLD has been defined as FLD in combination with one of the following: overweight (BMI > 25 kg/m^2^), T2DM, or at least two of the following signs of metabolic dysregulation: waist circumference ≥102/88 cm (males/females, respectively), blood pressure ≥130/85 mmHg (or treatment for hypertension), triglycerides ≥1.70 mmol/l, low HDL-C (<1.0 mmol/l for males or <1.3 for females, or specific medication), prediabetes (fasting glucose >5.6 mmol/l), homeostasis model assessment of insulin resistance score ≥2.5, or plasma high-sensitivity CRP > 2 mg/ml [16]. For the purpose of this study and considering the availability of parameters in our data, we considered the fulfillment of one metabolic component in the context of metabolic dysregulation sufficient to make a MAFLD diagnosis in conjunction with macrovesicular steatosis. The diseases, factors and metabolic variables included in the concept of MAFLD registered at the time of liver biopsy were overweight (BMI ≥ 25 kg/m^2^), T2DM (recorded or medicated), hypertension (recorded or medicated), dyslipidaemia (recorded, high triglycerides, low HDL-C or treatment) and elevated fasting glucose. Although our data included CRP values, these were not applicable as a MAFLD criteria in this context due to the influence of underlying liver diseases on the inflammation values.

### Statistics

Statistical analyses were performed with SPSS for Windows 27.0 (SPSS, Chicago, Illinois). Nonparametric statistical tests were used because variables were not normally distributed. Comparisons between the MAFLD and non-FLD groups were analyzed by Mann–Whitney *U* and *χ*^2^ or Fisher’s exact test for continuous and categorical variables, respectively. Fisher’s exact test was used for comparisons including small groups (*n* < 5). The performance of FIB-4, APRI and dAAR in detecting significant fibrosis (stages 2–3) was measured by area under the receiver operating characteristic analysis. A *P*-value <0.05 was considered statistically significant.

## Results

The study included 648 patients (Fig. 1); among these, 233 had AIH (including overlaps), 148 had PBC, 204 had PSC and 63 had toxic liver disease (Table 1). The etiology of toxic liver disease is reported in Supplementary Table 3, Supplemental Digital Content 1, http://links.lww.com/EJGH/B28. In the AIH group, 182 (78.1%) had AIH alone, 30 (12.9%) had AIH-PBC and 21 (9.0%) had AIH-PSC. No PBC/PSC overlaps were detected. Missingness rates for each variable are shown in Supplementary Table 2, Supplemental Digital Content 1, http://links.lww.com/EJGH/B28.

**Table 1.** Characteristics of patients with AIH, PBC, PSC and TOXIC

	AIH	PBC	PSC	TOXIC
	*N = *233	*N = *148	*N = *204	*N = *63
Age (years)	52.1 ± 17.5	57.3 ± 11.3	40.6 ± 16.5	48.7 ± 16.6
Women (*N*/%)	164/70.4	125/84.5	85/41.7	36/57.1
BMI (kg/m^2^)	27.2 ± 5.6	27.6.6 ± 5.4	25.3 ± 4.4	25.1 ± 4.4
Blood biochemistry				
Hemoglobin (g/l)	137 ± 14	134 ± 13	132 ± 18	135 ± 19
WBC (×10^9^/l)	6.6 ± 2.4	6.5 ± 2.1	6.9 ± 2.4	7.7 ± 3.7
Platelets (×10^9^/l)	242 ± 74	268 ± 71	281 ± 103	263 ± 109
CRP (mg/l)	7 ± 8	7 ± 8	15 ± 33	13 ± 19
ALT (U/l)	459 ± 590	77 ± 90	103 ± 108	729 ± 1419
AST (U/l)	359 ± 454	66 ± 62	69 ± 61	587 ± 1750
ALP (U/l)	167 ± 153	241 ± 238	259 ± 243	179 ± 147
Creatinine (μmol/l)	67 ± 22	70 ± 31	72 ± 18	80 ± 62
Glucose (mmol/l)	5.9 ± 1.0	6.0 ± 1.8	5.8 ± 1.2	5.8 ± 1.1
Cholesterol (mmol/l)	5.0 ± 1.4	6.0 ± 1.0	5.0 ± 1.4	5.1 ± 1.5
LDL-C (mmol/l)	2.9 ± 1.2	3.4 ± 1.0	2.8 ± 0.9	3.0 ± 1.4
HDL-C (mmol/l)	1.6 ± 0.8	1.8 ± 0.6	1.6 ± 0.6	1.3 ± 0.6
Triglycerides (mmol/l)	1.3 ± 0.7	1.4 ± 0.7	1.1 ± 0.6	1.8 ± 1.3
Noninvasive tests				
HSI	45.0 ± 58.4	39.4 ± 7.2	37.5 ± 7.4	40.3 ± 6.3
APRI	4.6 ± 6.4	0.73 ± 0.73	0.68 ± 0.74	14.9 ± 81.7
FIB-4	3.8 ± 4.1	1.7 ± 1.2	1.18 ± 0.99	5.9 ± 23.2
dAAR	3.4 ± 2.5	1.86 ± 1.47	0.96 ± 1.44	1.8 ± 1.3

Values are expressed as mean ± SD for continuous variables and *N*/% for sex.

AIH, autoimmune hepatitis; ALP, alkaline phosphatase; ALT, alanine aminotransferase; AST, aspartate aminotransferase; APRI, aspartate aminotransferase to platelet ratio index; CRP, C-reactive protein; dAAR, dynamic aspartate-to-alanine aminotransferase ratio; FIB-4, fibrosis-4 index; HDL-C, high-density lipoprotein cholesterol; HSI, hepatic steatosis index; LDL-C, low-density lipoprotein cholesterol; PBC, primary biliary cholangitis; PSC, primary sclerosing cholangitis; TOXIC, toxic liver disease; WBC, white blood cell count.

Steatosis was diagnosed in 101 (15.6%) patients. Of these, steatosis was attributable to MAFLD in 95 patients. Overall, there were only six patients with non-MAFLD steatosis. Among the MAFLD patients (*n* = 95), 85.3% (*n* = 81) had BMI > 25 kg/m², 44.2% (*n* = 42) had BMI > 30 kg/m², 22.1% (*n* = 21) had T2DM, 42.1% (*n* = 40) had prediabetes, 45.3% (*n* = 43) had hypertension and 63.2% (*n* = 60) had low HDL-C, high triglycerides, lipid lowering medication or combinations thereof. Eleven patients (11.6%) were diagnosed with MAFLD based solely on signs of metabolic dysregulation described above without obesity or T2DM; five of these had at least two components of metabolic dysregulation. The relative spectrum of the components of MAFLD in the liver diseases revealed that overweight together with dysmetabolism was predominant (Supplementary Figure 1, Supplemental Digital Content 1, http://links.lww.com/EJGH/B28).

**Fig. 2.**
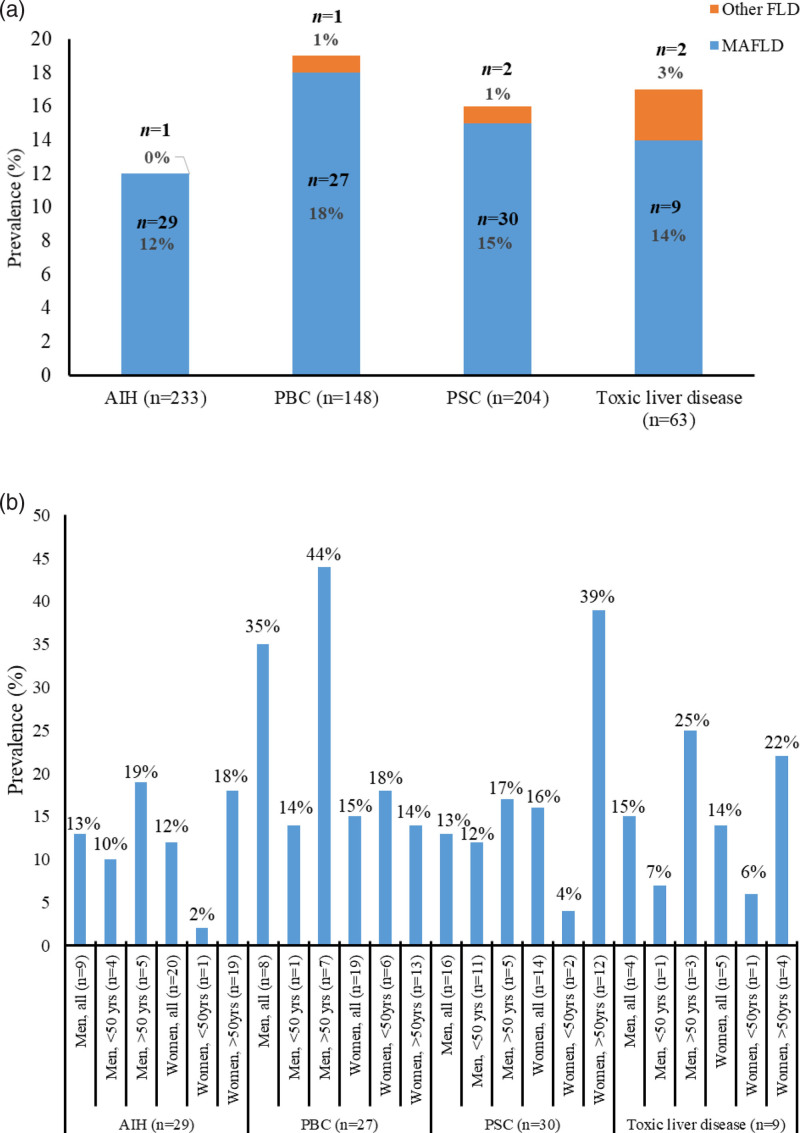
(a) Prevalence of MAFLD and other FLD in AIH, PBC, PSC, and toxic liver disease. (b) Disease-specific prevalence of MAFLD in men and women analyzed separately in two age groups with cutoff age 50 years. AIH, autoimmune hepatitis; FLD, fatty liver disease; MAFLD, metabolic-associated fatty liver disease; PBC, primary biliary cholangitis; PSC, primary sclerosing cholangitis.

The prevalence of MAFLD was 12.4% in AIH (including overlap), 18.2% in PBC, 14.7% in PSC and 14.3% in toxic liver disease (*P* = 0.483) (Fig. 2a).

The highest prevalence for PSC was observed in women aged >50 years (*P *= 0.065 for difference between sexes, Fig. 2b). Men >50 years with PBC had an MAFLD prevalence twice as high as other PBC age and sex groups (*P *= 0.04). In AIH and toxic liver disease patients, MAFLD prevalence was highest among patients aged >50 years, with no significant difference between the sexes.

Comparing MAFLD patients with matched non-FLD controls, liver enzymes were higher among matched controls than in AIH patients with MAFLD (Table 2). AST/ALT ratio was higher among matched controls in PSC patients with MAFLD.

**Table 2.** Comparison between patients with MAFLD and their disease-, age- and sex-matched non-FLD controls analysed separately for AIH, PBC, PSC and toxic liver disease

	AIH			PBC			PSC			Toxic liver disease		
	MAFLD *N* = 29	Non-FLD *N* = 30	*P*	MAFLD *N* = 27	Non-FLD *N* = 30	*P*	MAFLD *N* = 30	Non-FLD *N* = 30	*P*	MAFLD *N* = 9	Non-FLD *N* = 14	*P*
Age (years)	60.5 ± 11.5	61.0 ± 3.2	*0.585*	57.9 ± 9.6	58.6 ± 3.8	*0.299*	47.5 ± 15.1	46.3 ± 3.4	*0.204*	61.7 ± 19.1	58.5 ± 4.6	*0.277*
Women (*N*/%)	20/69.0	23/76.7	*0.568*	19/70.4	25/83.3	*0.346*	14/46.7	14/46.7	*1.000*	5/56	9/64.3	*1.000*
BMI (kg/m^2^)	28.1 ± 5.7	26.5 ± 5.6	*0.271*	31.6 ± 4.8	26.6 ± 4.7	***<0.001***	29.6 ± 4.4	24.3 ± 3.4	***<0.001***	29.0 ± 4.1	24.9 ± 4.8	***0.017***
Arterial hypertension (present) (*N*/%)	13/45	15/52	*0.793*	14/52	10/34	*0.280*	9/30	2/7	***0.042***	6/67	5/36	*0.214*
T2DM (present) (*N*/%)	9/31	7/23	*0.568*	6/22	1/3	***0.045***	3/10	1/3	*0.612*	3/33	0/0	***0.047***
Dyslipidemia (present) (*N*/%)	21/72	10/33	***0.004***	18/75	7/24	***<0.001***	13/45	1/3	***<0.001***	8/89	2/14	***<0.001***
Alcohol intake habit abstinent (*N*/%)	9/31	18/60	*0.076*	4/16	14/47	***0.034***	13/45	10/36	*0.425*	2/22	9/64	*0.120*
Social (*N*/%)	16/55	10/33		19/76	15/50		16/55	16/57		6/67	5/36	
Abuse (*N*/%)	4/14	2/7		2/8	1/3		0/0	2/7		1/11	0/0	
Cigarette consumption (present) (*N*/%)	8/28	2/7	***0.042***	8/30	3/10	*0.093*	7/24	2/7	*0.078*	3/33	1/7	*0.260*
Blood biochemistry												
Hemoglobin (g/l)	137 ± 14	140 ± 14	*0.676*	140 ± 12	134 ± 11	*0.064*	138 ± 15	136 ± 16	*0.803*	147 ± 6	128 ± 24	***0.025***
WBC (×10E9/l)	6.8 ± 3.0	6.6 ± 2.2	*0.927*	7.1 ± 1.6	6.2 ± 1.7	*0.062*	7.5 ± 2.8	6.1 ± 2.2	*0.069*	8.5 ± 4.5	7.9 ± 5.2	*0.488*
Platelets (×10E9/l)	228 ± 68	248 ± 86	*0.471*	274 ± 83	259 ± 51	*0.712*	279 ± 66	281 ± 109	0.322	201 ± 80	277 ± 112	*0.298*
CRP (mg/l)	12.3 ± 14.0	6.5 ± 7.3	*0.055*	7.4 ± 5.8	8.5 ± 8.0	*0.936*	22.8 ± 46.8	4.5 ± 7.0	*0.089*	16 ± 14	10 ± 17	*0.278*
ALT (U/l)	319 ± 536	568 ± 637	***0.024***	78 ± 50	92 ± 164	*0.447*	86 ± 56	73 ± 64	*0.139*	1716 ± 3579	435 ± 437	*0.714*
AST (U/l)	274 ± 411	541 ± 599	***0.036***	61 ± 31	68 ± 92	*0.271*	52 ± 24	59 ± 48	*0.551*	1915 ± 4695	331 ± 406	*0.918*
AST/ALT-ratio	1.03 ± 0.44	0.98 ± 0.57	*0.354*	0.83 ± 0.32	1.26 ± 1.90	*0.666*	0.68 ± 0.25	1.05 ± 0.45	***0.002***	0.67 ± 0.30	0.70 ± 0.23	*0.616*
ALP (U/l)	115 ± 65	180 ± 153	***0.013***	179 ± 96	214 ± 172	*0.612*	171 ± 89	221 ± 219	*0.976*	136 ± 63	183 ± 134	*0.823*
Creatinine (μmol/l)	64 ± 10	61 ± 15	*0.348*	68 ± 14	74 ± 29	*0.632*	69 ± 14	73 ± 14	*0.224*	112 ± 102	75 ± 43	*0.271*
Glucose (mmol/l)	6.0 ± 1.0	5.6 ± 0.7	*0.171*	6.2 ± 1.5	5.9 ± 2.0	***0.025***	5.9 ± 0.8	5.8 ± 1.5	*0.177*	6.4 ± 1.3	5.5 ± 0.9	***0.048***
Cholesterol (mmol/l)	5.1 ± 1.2	4.8 ± 1.0	*0.376*	5.8 ± 1.3	5.7 ± 0.9	*0.609*	5.1 ± 1.3	5.4 ± 1.2	*0.400*	4.6 ± 1.1	5.2 ± 1.8	*0.396*
LDL-C (mmol/l)	3.3 ± 1.0	2.7 ± 1.0	***0.049***	3.6 ± 1.1	3.3 ± 0.8	*0.292*	3.0 ± 0.7	3.1 ± 0.7	*0.621*	2.8 ± 1.2	3.2 ± 1.4	*0.616*
HDL-C (mmol/l)	1.3 ± 0.5	1.5 ± 0.7	*0.231*	1.7 ± 0.6	2.0 ± 0.6	***0.023***	1.4 ± 0.5	1.8 ± 0.7	***0.023***	1.3 ± 0.2	1.6 ± 0.7	*0.063*
Triglycerides (mmol/l)	1.5 ± 0.8	1.2 ± 0.6	*0.313*	1.8 ± 0.8	1.1 ± 0.5	***<0.001***	1.3 ± 0.5	1.2 ± 0.9	*0.067*	2.8 ± 1.2	3.2 ± 1.4	*0.526*
Non-invasive tests												
HSI	39.8 ± 7.9	38.6 ± 7.1	*0.617*	43.7 ± 6.8	38.4 ± 7.3	***0.017***	44.1 ± 6.3	35.1 ± 6.1	***<0.001***	44.6 ± 6.2	38.7 ± 6.0	***0.024***
APRI	3.9 ± 6.2	7.9 ± 10.0	*0.051*	0.64 ± 0.37	0.80 ± 1.17	*0.753*	0.49 ± 0.25	0.57 ± 0.42	*0.899*	80.9 ± 220	4.8 ± 6.4	*0.877*
FIB4 index	4.6 ± 5.2	6.1 ± 6.4	*0.436*	1.55 ± 0.79	1.76 ± 1.16	*0.860*	1.11 ± 0.70	1.46 ± 0.99	*0.108*	24.5 ± 62.6	4.1 ± 4.0	*0.920*
dAAR	3.69 ± 2.33	4.53 ± 2.85	*0.354*	2.00 ± 1.05	1.57 ± 0.33	*0.296*	1.16 ± 1.07	1.15 ± 1.35	*0.927*	11.0 ± 22.3	3.2 ± 2.2	*0.616*

Values are expressed as mean ± SD for continuous and *N*/% for categorical variables. *P*-value indicates Mann–Whitney *U* comparison between MAFLD and non-FLD subgroups or Chi-square or Fisher test in categorical variables.

Statistically significant *P* values are presented in bold italics.

AIH, autoimmune hepatitis; ALP, alkaline phosphatase; ALT, alanine aminotransferase; AST, aspartate aminotransferase; APRI, aspartate aminotransferase to platelet ratio index; dAAR, dynamic aspartate-to-alanine aminotransferase ratio; FIB-4, fibrosis-4 index; FLD, fatty liver disease; HDL-C, high-density lipoprotein cholesterol; HSI, hepatic steatosis index; LDL-C, low-density lipoprotein cholesterol; MAFDL, metabolic associated fatty liver disease; PBC, primary biliary cholangitis; PSC, primary sclerosing cholangitis; T2DM, type-2 diabetes; TOXIC, toxic liver disease; WBC, white blood cell count.

### Alcohol consumption and smoking

Distribution of MAFLD and matched non-FLD controls categorized according to alcohol consumption and smoking habits are shown in Table 2 and Supplementary Figure 2, Supplemental Digital Content 1, http://links.lww.com/EJGH/B28. Alcohol consumption was more common among MAFLD patients than matched non-FLD controls within the AIH (69 vs 40%, *P *= 0.04) and PBC groups (84 vs 53%, *P *= 0.02). In the AIH group, current smoking was more common among MAFLD patients than non-FLD controls (27.6 vs 6.7%, *P* = 0.04).

### Liver histopathology

Comparison of the histopathological characteristics between the MAFLD and the control groups according to liver diagnosis is shown in Table 3. Fibrosis was more prevalent in the MAFLD group among patients with toxic liver disease (*P* = 0.01). Otherwise, inflammation, fibrosis, lobular and interface activity, and cytokeratin 7 stain showed a similar distribution among patients with MAFLD and non-FLD patients in all diseases observed. Steatohepatitis was observed in 13 (13.7%) patients in the MAFLD group. Within the MAFLD group, the prevalence of steatohepatitis in the different diagnosis groups were the following: 24.7% (*n* = 7) in AIH and overlap, 11.1% (*n* = 3) in PBC, 6.7% (*n* = 2) in PSC and 11.1% (*n* = 1) in toxic liver disease (*P *= 0.25). Among patients with AIH, PBC or PSC, mean microvesicular steatosis in the control groups varied between 2 and 8% [standard deviations (SD) 7–14] and in the MAFLD groups between 28 and 30% (SDs 23–26). In toxic liver disease, mean percentage of microvesicular fat was 16% (SD 30) for the control group and 18% (SD 18) for the MAFLD group.

**Table 3.** Histopathologic characteristics for patients with steatosis and nonmatched controls

Diagnosis	AIH and overlap^a^			PBC			PSC			Toxic liver disease		
	MAFLD *N* = 29	Controls *N* = 203	*P*-value	MAFLD *N* = 27	Controls *N* = 120	*P*-value	MAFLD *N* = 30	Controls *N* = 172	*P*-value	MAFLD *N* = 9	Controls *N* = 52	*P*-value
Inflammation												
Grade												
0	3 (10%)	23 (11%)	*0.54*	6 (22%)	34 (28%)	*0.50*	21 (70%)	106 (62%)	*0.82*	3 (33%)	16 (31%)	*0.96*
1	8 (28%)	42 (21%)		16 (59%)	59 (42%)		9 (30%)	51 (30%)		2 (22%)	9 (17%)	
2	8 (28%)	46 (23%)		4 (15%)	17 (14%)		0	8 (5%)		2 (22%)	8 (15%)	
3	9 (31%)	91 (45%)		0	9 (8%)		0	3 (2%)		2 (22%)	15 (29%)	
Fibrosis^b^												
Stage												
0	6 (21%)	68 (34%)	*0.47*	6 (22%)	37 (31%)	*0.72*	17 (57%)	82 (48%)	*0.80*	2 (22%)	37 (71%)	*0.01*
1	11 (38%)	58 (29%)		15 (56%)	55 (46%)		6 (20%)	48 (28%)		4 (44%)	8 (15%)	
2	9 (31%)	59 (29%)		4 (15%)	23 (19%)		5 (17%)	28 (16%)		2 (22%)	4 (8%)	
3	3 (10%)	16 (8%)		1 (4%)	4 (3%)		2 (7%)	10 (6%)		1 (11%)	1 (2%)	
Significant fibrosis												
Stages												
0–1	17 (59%)	126 (62%)	*0.67*	21 (78%)	92 (77%)	*0.70*	23 (77%)	130 (76%)	*0.93*	6 (67%)	45 (87%)	*0.09*
2–3	12 (41%)	75 (37%)		5 (19%)	27 (23%)		7 (23%)	38 (22%)		3 (33%)	5 (10%)	
Advanced fibrosis												
Stages												
0–2	26 (90%)	185 (91%)	*0.66*	25 (93%)	115 (95%)	*1.0*	28 (93%)	158 (93%)	*1.0*	8 (89%)	49 (94%)	*0.28*
3	3 (10%)	16 (8%)		1 (4%)	4 (3%)		2 (7%)	10 (6%)		1 (11%)	1 (2%)	
Lobular activity												
0	6 (21%)	53 (26%)	*0.53*	9 (33%)	63 (53%)	*0.09*	24 (80%)	139 (81%)	*1.0*	4 (44%)	19 (37%)	*0.77*
1	13 (45%)	63 (31%)		17 (63%)	48 (40%)		5 (17%)	30 (17%)		1 (11%)	14 (27%)	
2	10 (35%)	80 (39%)		1 (4%)	9 (8%)		0	3 (2%)		4 (44%)	18 (35%)	
3	0	6 (3%)		0	0		0	0		0	1 (2%)	
Interface activity												
0	6 (21%)	28 (14%)	*0.28*	7 (26%)	40 (33%)	*0.70*	21 (70%)	118 (69%)	*0.59*	3 (33%)	22 (42%)	*0.95*
1	6 (21%)	48 (24%)		16 (59%)	57 (48%)		9 (30%)	44 (26%)		3 (33%)	12 (23%)	
2	12 (41%)	68 (34%)		4 (15%)	18 (15%)		0	9 (5%)		2 (22%)	11 (21%)	
3	4 (14%)	59 (29%)		0	5 (4%)		0	1 (1%)		1 (11%)	7 (14%)	
Steatosis												
Macro												
<5%	0	203 (100%)		0	120 (100%)		0	172 (100%)		0	52 (100%)	
5–35%	24 (83%)	0		24 (89%)	0		26 (87%)	0		8 (89%)	0	
35–65%	4 (14%)	0		1 (4%)	0		3 (10%)	0		0	0	
>65%	1 (3%)	0		2 (7%)	0		1 (3%)	0		1 (11%)	0	
Micro												
<5%	1 (3%)	164 (81%)	*<0.001*	3 (11%)	100 (83%)	*<0.001*	2 (7%)	148 (86%)	*<0.001*	1 (11%)	37 (71%)	*<0.001*
5–35%	21 (72%)	34 (17%)		19 (70%)	18 (15%)		18 (60%)	20 (12%)		7 (78%)	14 (27%)	
35–65%	6 (21%)	4 (2%)		3 (11%)	1 (1%)		9 (30%)	4 (2%)		1 (11%)	0	
>65%	1 (3%)	0		2 (7%)	1 (1%)		1 (3%)	0		0	1 (2%)	
Steatohepatitis												*0.27*
0	22 (76%)	198 (98%)	*<0.001*	24 (89%)	119 (99%)	*0.02*	28 (93%)	172 (100%)	*0.02*	8 (89%)	47 (90.4)	
1	6 (21%)	4 (2%)		2 (7%)	1 (1%)		1 (3%)	0		0	0	
2	0	1 (1%)		1 (4%)	0		1 (3%)	0		01 (11%)	1 (2%)	
3	1 (3%)	0		0	0		0	0		0	0	
CK7+^c^												
0				7 (26%)	26 (22%)	*0.53*	11 (37%)	42 (24%)	*0.32*			
1				6 (22%)	44 (37%)		6 (20%)	36 (21%)				
2				2 (7%)	17 (14%)		1 (3%)	22 (13%)				
3				0	0		0	2 (1%)				

Values given for *N* and (%).

Statistically significant *P* values are presented in italics.

AIH, autoimmune hepatitis; MAFLD, metabolic-associated fatty liver disease; PBC, primary biliary cirrhosis; PSC, primary sclerosing cholangitis.

a Including overlap disease with PBC or PSC.

b Patients with stage 4 fibrosis in index biopsy were excluded.

c Hepatocytes positive for cytokeratin 7, graded 0–3.

When MAFLD patients were compared with matched controls, interface activity was more severe among the matched controls in the AIH group. Eighty-seven percent of the matched controls had moderate/severe (grade 2–3) interface activity, while only 57% of MAFLD patients had moderate/severe interface activity (*P *= 0.06) (Supplementary Table 4, Supplemental Digital Content 1, http://links.lww.com/EJGH/B28). In the AIH group, inflammation was more severe among matched controls (*P* = 0.02) than in MAFLD patients. Further analysis revealed a difference in both interphase and lobular activity without statistical significance (*P *= 0.06 and *P *= 0.30, respectively). Otherwise, the comparison between MAFLD patients and matched controls did not reveal any significant differences in inflammation, fibrosis or lobular/interface activity (or cytokeratin 7 positive hepatocytes for PBC and PSC) for any of the diagnosis groups.

### Performance of noninvasive tests and ultrasound

Although HSI scores were higher for MAFLD patients than matched controls for patients with PBC, PSC or toxic liver disease (Table 2), only the matched controls in the PSC group had a median score (33.5) below the upper cutoff to detect steatosis (36). The median for MAFLD patients in the PSC group was above the cutoff (43.8).

In the combined sample (AIH, PSC, PBC and toxic liver disease together), AUC values of NITs (FIB-4, APRI and dAAR) for discriminating F2–3 from F0–1 did not differ significantly between the non-FLD group and the MAFLD group (*P *= 0.85–0.98) (Supplementary Table 5, Supplemental Digital Content 1, http://links.lww.com/EJGH/B28 for all NIT data).

Ultrasound showed signs of fatty liver in 72 patients in the matched MAFLD and non-FLD groups; of these, 15 (21%) were categorized as non-FLD by histology. Ultrasound failed to detect ≥5% macrovesicular steatosis in 40% of the 95 patients with biopsy-confirmed steatosis. Sensitivity and specificity of ultrasound to detect ≥5% macrovesicular steatosis was 57.4 and 87.2%, respectively. The sensitivity of ultrasound to detect >35% macrovesicular steatosis was higher (80.0%); specificity was 72.7%. AUC for ultrasound to detect ≥5% macrovesicular steatosis was 0.72 (95% CI 0.66–0.79). Additional performance measures of ultrasound are shown in Supplementary Table 6, Supplemental Digital Content 1, http://links.lww.com/EJGH/B28 and Supplementary Figure 3, Supplemental Digital Content 1, http://links.lww.com/EJGH/B28.

## Discussion

The main new findings of this cross-sectional real-world liver biopsy study were the following: 12.4–18.2% of patients with other primary liver disease (AIH, PSC, PBC or toxic liver disease) had coexistent FLD; the vast majority (94%) of the FLD cases were categorized as MAFLD; and patients with MAFLD did not seem to have more severe histopathological findings than their non-FLD counterparts within each diagnosis group.

The prevalence of liver steatosis and MAFLD in this patient sample were lower than in a recently published meta-analysis [17]. This may be due in part to patient awareness of liver disease, which may have motivated them to adopt a healthier lifestyle. This possibility, however, does not entirely explain this observation, as for some patients, particularly those with AIH, the biopsy was taken at the beginning of the diagnostic procedure. Overall, data on metabolic syndrome among patients with autoimmune liver diseases are lacking. The proportion of steatosis patients that met the MAFLD criteria was high (94%). This suggests that metabolic factors are essential in development of FLD also in the context of other primary liver diseases.

MAFLD, and especially NASH, are known causes of liver cirrhosis and its complications [18]. Clinical management of patients with coexisting MAFLD and other primary liver disease should thus also focus on treatment of MAFLD [19,20], and the known risk factors of MAFLD. In the present study, we revealed for the first time the relative spectrum of components of MAFLD in a liver-biopsy cohort. Overweight together with dysmetabolism played a central role in each liver disease and were the most predominant in toxic liver disease. Overall, this finding emphasizes the importance of overweight/obesity, hypertension, dyslipidaemia and elevated serum glucose in clinical practice. In particular, T2DM is linked to progression of fibrosis in NAFLD [18]. The effect of MAFLD on disease progression in other primary liver disease is unknown, and further studies are needed to examine any possible additive effect on liver fibrosis progression and risk for liver-related outcomes. The finding of the present study that the AIH patients with MAFLD had lower liver enzyme values than the respective non-FLD controls is a novel one. It is difficult to explain based on the present data and it deserves more research in the future. Although NAFLD did not alter disease progression based on NITs in patients with PBC in one study [21], repeated NITs may not correlate well with actual disease progression in FLD [22]. PBC is linked to a less atherogenic lipid profile and to a lower prevalence of FLD when compared with other liver diseases [23]. In the present study, however, FLD prevalence among PBC patients did not clearly differ from the FLD prevalence in the other liver disease groups. Animal models of PSC have shown associations between NAFLD and cholangitis progression and PSC-related malignancies [24]. Previous studies have shown NAFLD to be associated with toxic liver disease [25]. Furthermore, Medina-Caliz *et al*. have shown that in the context of drug-induced liver disease, metabolic factors are associated with chronicity of the liver injury [26], which is consistent with the findings of our study showing more severe fibrosis in the toxic liver disease group with MAFLD than non-FLD controls.

The inflammation grade or fibrosis stage by histopathology was similar between MAFLD and non-FLD groups as analyzed separately by liver disease diagnosis except for toxic liver disease, where fibrosis was more severe among MAFLD patients. The lowest proportion of fibrosis was observed in the toxic liver disease non-FLD group, which is consistent with the acute presentation often seen in toxic liver disease [26]. The lack of chronic fibrinogenic factors for at least a portion of the patients in the toxic liver disease non-FLD group would explain the observed difference in fibrosis, with MAFLD, to some extent, contributing to the increased fibrosis in MAFLD patients. Non-FLD AIH patients had more severe interface activity than AIH patients with MAFLD, which is consistent with the observed higher liver enzyme levels among the non-FLD AIH controls.

NITs (FIB-4, APRI and dAAR) performed poorly in discriminating significant fibrosis in the present study, which is partly explained by the exclusion of cirrhosis patients as part of the study design. Cirrhosis patients were excluded as liver fat can disappear following cirrhosis development, which would thus confound prevalence analyses. The performance of NITs, however, did not seem to differ between MAFLD and non-FLD patients. This finding suggests that the presence of other liver disease does not limit the applicability of NITs for detection of fibrosis. Median HSI values were above the lower cutoff, which limits the use of HSI to exclude FLD in patients with other primary liver disease. HSI, however, is applicable regardless of confounding factors, such as alcohol consumption habits [27]. Ultrasound has generally been used to detect liver steatosis. In this regard, it is interesting to note that ultrasound steatosis and histopathological steatosis did not perfectly match in the present study.

A strength of this study is that liver disease diagnoses were biopsy-verified and structured reporting of histopathological findings was used. Furthermore, the patients were carefully examined at specialized centres. At the Helsinki University Hospital district, liver biopsy is routinely performed as part of the baseline clinical evaluation of PSC or PBC (in AIH the biopsy is included routinely worldwide in this context). Our study cohort is a representative sample of the Helsinki University Hospital district (1.7 million inhabitants), which reduces the risk of selection bias.

A limitation of our study is that real-world hospital records lack some parameters, such as waist circumference, high-sensitive CRP and Homeostasis Model Assessment for Insulin Resistance, which are required to evaluate all MAFLD criteria or to calculate some indices of steatosis or fibrosis. Fortunately, the information technology managements were able to search for clinical data from hospital records of large patient cohorts. In contrast, the time-consuming manual browsing by a researcher enabled more accurate and complete data collection. The number of patients retrieved by this approach, however, tends to be lower, and this was the case in our comparison analysis of MAFLD patients versus matched non-FLD patients. The size and the accuracy of the clinical data in these groups of about 100 patients each, however, are representative. Furthermore, the remaining >400 patients, from which the data were obtained almost solely by information technology management, have been characterized sufficiently well to draw conclusions between the MAFLD and non-FLD groups. The present study was designed, and the data were collected and analysed according to the MAFLD criteria, which were later replaced by the definition of metabolic dysfunction associated steatotic liver disease (MASLD) [28]. The difference between the definitions of MAFLD and MASLD have no practical influence on the interpretation of the present data [29].

In conclusion, the prevalence of FLD in AIH, PBC, PSC and toxic liver disease patients was lower than previously reported but comparable with each other. The vast majority of FLD is due to MAFLD. There were no major differences in histopathological characteristics between MAFLD patients and non-FLD patients. Further studies to assess the prognostic value of coexisting MAFLD are warranted.

## Acknowledgements

O.D. was supported by grants from the Helsinki University Hospital Research Fund, the Sigrid Jusélius Foundation, the Mary and Georg Ehrnrooth Foundation, and by the Academy of Finland. M.J.N. was supported by grants from the Sigrid Jusélius Foundation, the Mary and Georg Ehrnrooth Foundation, and by the Academy of Finland. T.V. was supported by grants from the Helsinki University Hospital Research Fund and Cancer Foundation Finland. J.A. was supported by grants from the Helsinki University Hospital Research Fund, the HUS Diagnostic Centre research fund, and Cancer Foundation Finland. F.A. was supported by the Academy of Finland (grant #338544), the Sigrid Jusélius Foundation, and Finska Läkaresällskapet.

All authors contributed to the design of the study, to the analysis of the results and to the writing of the manuscript.

### Conflicts of interest

There are no conflicts of interest.

## Supplementary Material


