# Supplementary material for: Coexistence of metabolic-associated fatty liver disease and autoimmune or toxic liver disease
Source: Eur J Gastroenterol Hepatol. 2024 Apr 26;36(7):961–9. doi: 10.1097/MEG.0000000000002785 (PMC11136267; doi:10.1097/MEG.0000000000002785)
Supplement: Supplementary file 1 [file ejgh-36-961-s001.pdf]

Supplementary Figure 1

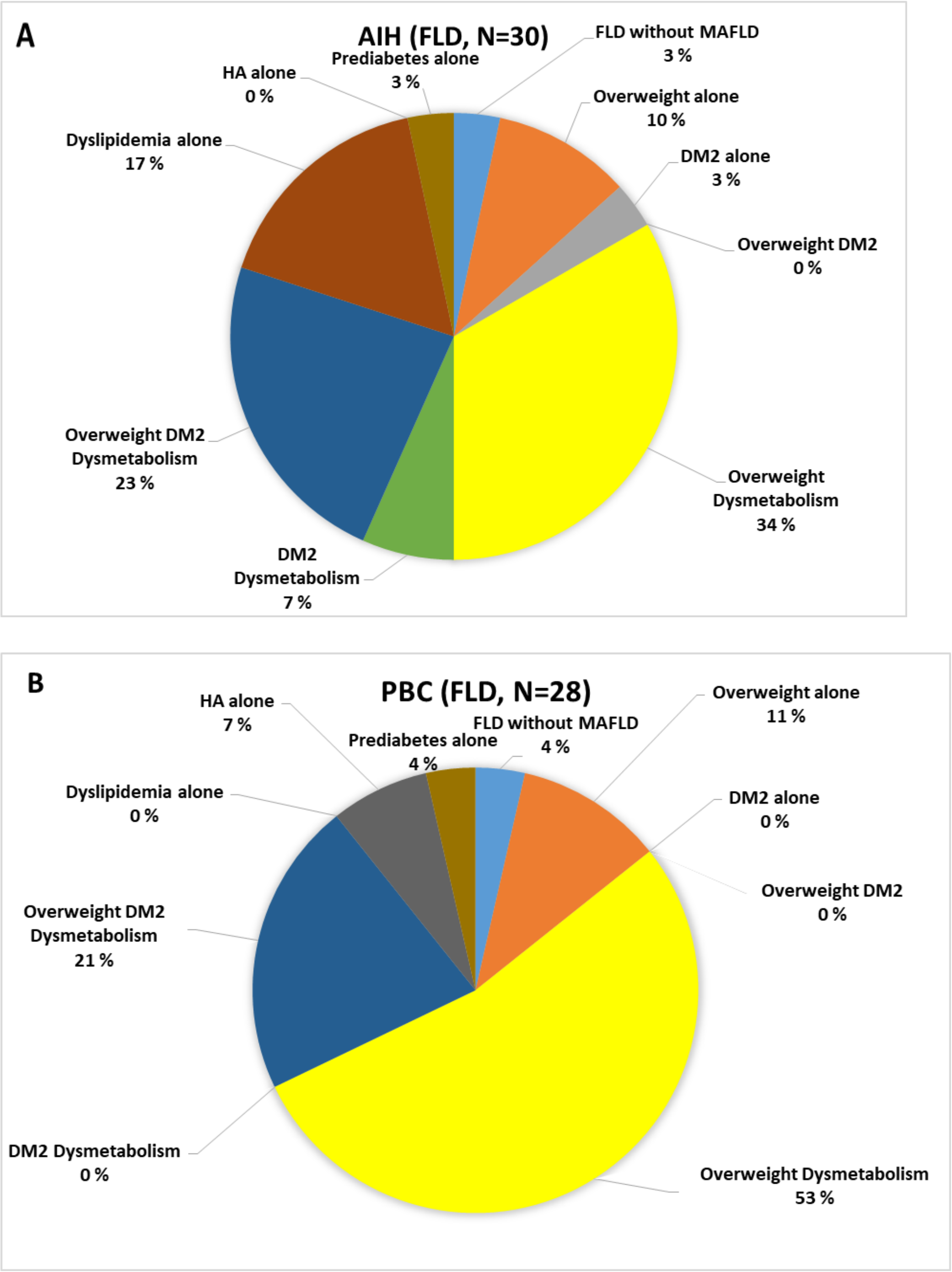

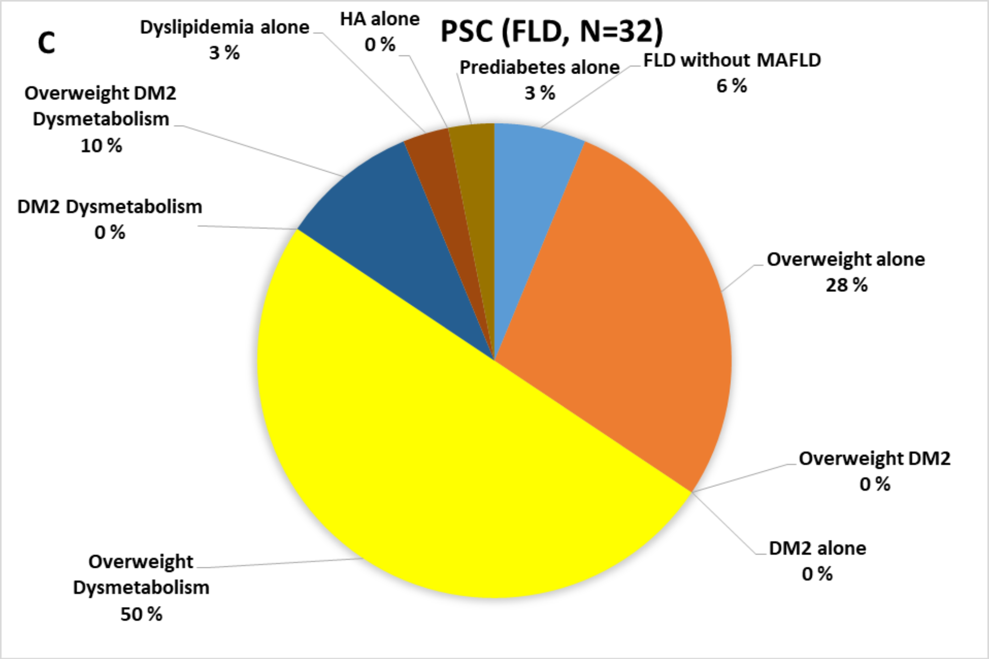

C
PSC (FLD, N=32)
Dyslipidemia alone
3 %
HA alone
0 %
Prediabetes alone
3 %
FLD without MAFLD
6 %
Overweight DM2
Dysmetabolism
10 %
DM2 Dysmetabolism
0 %
Overweight alone
28 %
Overweight DM2
0 %
DM2 alone
0 %
Overweight
Dysmetabolism
50 %

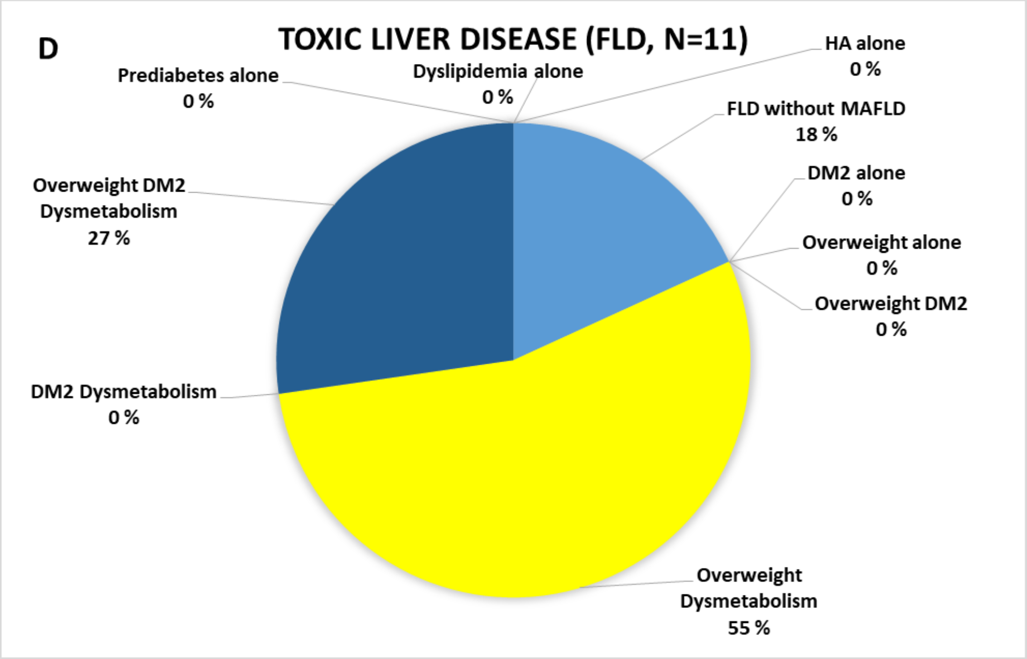

D
TOXIC LIVER DISEASE (FLD, N=11)
HA alone
0 %
Prediabetes alone
0 %
Dyslipidemia alone
0 %
FLD without MAFLD
18 %
Overweight DM2
Dysmetabolism
27 %
DM2 alone
0 %
Overweight alone
0 %
Overweight DM2
0 %
DM2 Dysmetabolism
0 %
Overweight
Dysmetabolism
55 %

Supplementary Figure 2

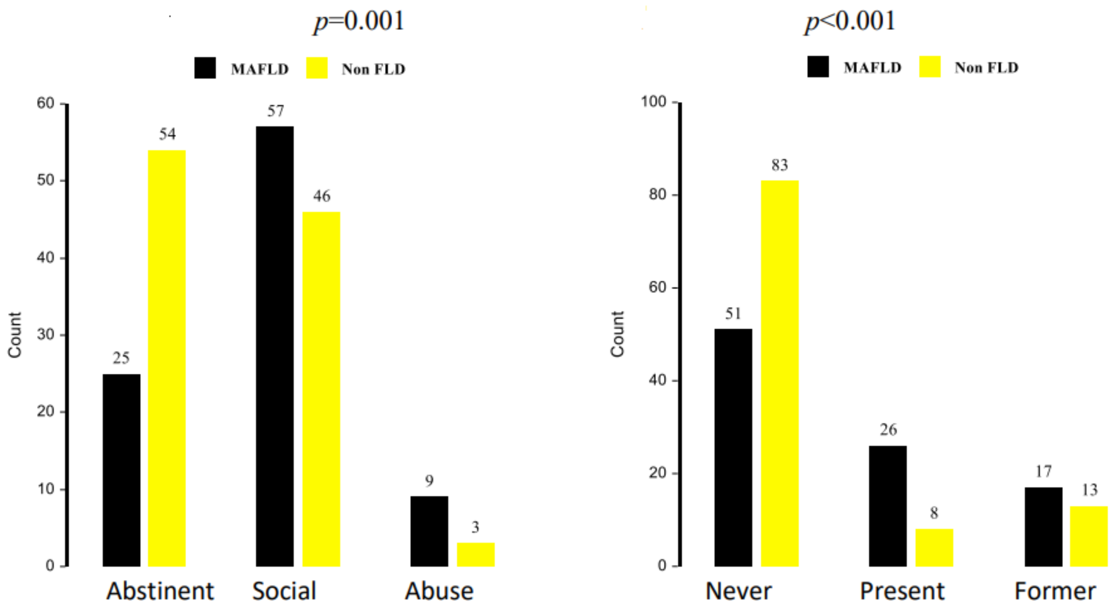

Supplementary Figure 3

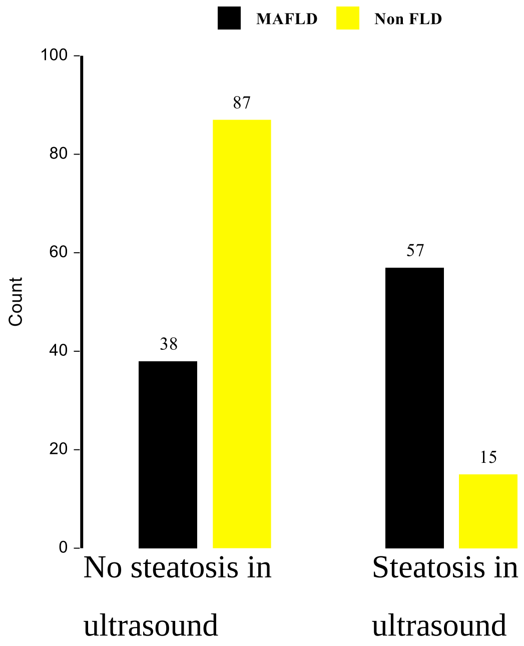

Supplementary Figure legends

Supplementary figure 1 legend

The pie charts reveal relative spectrum of the components of metabolic associated fatty liver disease (MAFLD) in autoimmune hepatitis (AIH) (A), primary biliary cholangitis (PBC) (B), primary sclerosing cholangitis (PSC) (C) and toxic liver disease (D). FLD, fatty liver disease.

Supplementary figure 2 legend

Absolute distribution of metabolic-associated fatty liver disease (MAFLD) and non-fatty liver disease (non-FLD) of the matched liver biopsy study cohorts in categorized groups of alcohol consumption (A), smoking habits (B).

Supplementary figure 3 legend

Patients without and with steatosis finding in ultrasound. Shown separately for metabolic-associated fatty liver disease (MAFLD) patients and non-fatty liver disease (non-FLD) patients. *I.e.,* patients with histological steatosis and patients without histological steatosis.

Supplementary Table 1. The score equations.

Fibrosis-4 index (FIB-4)

= (AGE * AST) / ((platelets) * square root (ALT))

AST to platelet ratio index (APRI)

= (AST/upper limit of the normal AST range) * 100 / platelets

Hepatic steatosis index (HSI)

= 8 * (ALT/AST ratio) + BMI (+2, if female; +2, if diabetes mellitus)

Dynamic aspartate-to-alanine aminotransferase ratio (dAAR) (Microsoft Excel)

= (-10,129915)+(0,039811813*AGE)+(0,25387407*ALT)-(0,0023607234*MAX(0;ALT-11)^3)+(0,0079492072*MAX(0;ALT-17)^3)-(0,0076811579*MAX(0;ALT-22)^3)+(0,0021985068*MAX(0;ALT-30)^3)-(0,00010583268*MAX(0;ALT-58)^3)+(3,5333535*(ASTALT))-(7,3473709*MAX(0;ASTALT-0,63)^3)+(32,911587*MAX(0;ASTALT-0,92)^3)-(44,937707*MAX(0;ASTALT-1,14)^3)+(21,786619*MAX(0;ASTALT-1,14)^3)-(2,4131284*MAX(0;ASTALT-2,13)^3)

AGE = age in years, AST = aspartate aminotransferase (U/L), platelets = platelet count (10^9 /L) ALT = alanine aminotransferase (U/L), BMI = body mass index, ASTALT = ratio of AST to ALT

Supplementary Table 2.

Absolute and relative amounts of missing data in the whole study sample (*N*=648) and in the FLD (*N*=101) group.

	**Total sample Missing values** (*N*)	**Total sample Percentage of missing values** (%)	**FLD group Absolute and relative amounts of missing values (*N* ≈ %)**
Age (years)	0	0	0
Sex	0	0	0
Liver disease	0	0	0
Liver histopathology	0	0	0
Listing of all recorded diagnosis during the study period	0	0	0
Liver ultrasound	391	60	0
BMI (kg/m^2)	189	29	2
Arterial blood pressure	392	60	0
Diagnosis of DM2	0	0	0
Diagnosis of dyslipidemia[1]	349	54	4
Alcohol intake habit	395	61	5
Smoking	392	60	3
**Non-invasive tests**			
HSI	240	37	10
APRI	91	14	10
FIB4 index	85	13	10
dAAR	76	12	9

**Biochemical examinations**			
Hemoglobin	13	2	1
White blood cell count	13	2	1
Platelets	11	2	1
CRP (mg/L)	164	25	30
ALT (U/L)	24	4	6
AST (U/L)	79	12	9
GT (U/L)	327	50	44
ALP (U/L)	28	4	7
Albumin	255	39	44
Tromboplastin time	21	3	6
Cholesterol	191	29	13
LDL-cholesterol	279	43	22
HDL-cholesterol	287	44	25
Triglycerides	282	44	26
Creatinine	155	24	29
Ferritin	465	72	84
Glucose	457	71	13

[1]Diagnosis of dyslipidemia was based on statin medication in 22 FLD-patients lacking the data on the lipid values at the time of the data collection (±3 months the liver biopsy).

Supplementary Table 3.

Characteristics of patients with toxic liver disease by etiology and subgrouping by metabolic associated fatty liver disease (MAFLD), fatty liver disease (FLD) without MAFLD and non-FLD.

Etiologic factor	MAFLD<br>*N*=9	FLD without MAFLD<br>*N*=2	Non-FLD<br>*N*=52
Acetaminophen	1		1
Amoxicillin		1	1
Amoxicillin+albendazole			1
Amoxicillin+clavulanic acid			4
Amoxicillin+disulfiram			1
Amoxicillin+doxycycline			1
Anabolic steroids			2
Cephalexin			1
Clindamycin			1
Disulfiram			1
Doxycycline			1
Ezetimibe			1
Herbal product	1		6
Imatinib			1
IV-drugs			1
Methotrexate		1	
Mitoxantrone			1
Moxifloxacin			1
NSAID	1		1
NSAID+acetaminophen			1
Oral contraceptives			1
Pembrolizumab	1		
Ramipril			2
Ritonavir			1
Simvastatin	2		1
Trazodone	1		
Unknown	2		18
Valproate			1

IV, intravenous; NSAID, non-steroidal anti-inflammatory drug

Supplementary Table 4.

Histopathologic characteristics for patients with steatosis and matched controls.

Diagnosis		AIH and overlap*			PBC			PSC			Toxic liver disease		
		MAFLD N=29	Controls N=30	*p*-value	MAFLD N=27	Controls N=30	*p*-value	MAFLD N=30	Controls N=30	*p*-value	MAFLD N=9	Controls N=14	*p*-value
Inflammation													
Grade	0	3 (10%)	2 (7%)	*0.02*	6 (22%)	9 (30%)	*0.22*	21 (70%)	22 (73%)	*0.11*	3 (33%)	3 (25%)	*0.82*
	1	8 (28%)	1 (3%)		16 (59%)	13 (43%)		9 (30%)	5 (17%)		2 (22%)	4 (33%)	
	2	8 (28%)	8 (27%)		4 (15%)	4 (13%)		0	3 (10%)		2 (22%)	1 (8%)	
	3	9 (31%)	19 (63%)		0	4 (13%)		0	0		2 (22%)	4 (33%)	
Fibrosis**													
Stage	0	6 (21%)	8 (27%)	*0.95*	6 (22%)	10 (33%)	*0.72*	17 (57%)	19 (63%)	*0.85*	2 (22%)	10 (77%)	*0.06*
	1	11 (38%)	10 (33%)		15 (56%)	13 (43%)		6 (20%)	7 (23%)		4 (44%)	2 (15%)	
	2	9 (31%)	8 (27%)		4 (15%)	5 (17%)		5 (17%)	3 (10%)		2 (22%)	1 (8%)	
	3	3 (10%)	4 (13%)		1 (4%)	2 (7%)		2 (7%)	1 (3%)		1 (11%)	0	
Significant fibrosis													
Stages	0-1	17 (59%)	18 (60%)	*0.91*	21 (78%)	23 (77%)	*0.71*	23 (77%)	26 (87%)	*0.51*	6 (67%)	12 (92%)	*0.26*
	2-3	12 (41%)	12 (40%)		5 (19%)	7 (23%)		7 (23%)	4 (13%)		3 (33%)	1 (8%)	
Advanced fibrosis													
Stages	0-2	26 (90%)	26 (87%)	*1.0*	25 (93%)	28 (93%)	*1.0*	28 (93%)	29 (97%)	*1.0*	8 (89%)	13 (100%)	*0.41*
	3	3 (10%)	4 (13%)		1 (4%)	2 (7%)		2 (7%)	1 (3%)		1 (11%)	0	

Lobular activity													
	0	6 (21%)	5 (17%)	*0.30*	9 (33%)	14 (47%)	*0.18*	24 (80%)	28 (93%)	*0.20*	4 (44%)	5 (36%)	*1.0*
	1	13 (45%)	8 (28%)		17 (63%)	12 (40%)		5 (17%)	2 (7%)		1 (11%)	3 (21%)	
	2	10 (35%)	16 (55%)		1 (4%)	4 (13%)		0	0		4 (44%)	5 (36%)	
	3	0	0		0	0		0	0		0	1 (7%)	
Interface activity													
	0	6 (21%)	2 (7%)	*0.06*	7 (26%)	12 (40%)	*0.28*	21 (70%)	22 (73%)	*0.14*	3 (33%)	7 (50%)	*0.86*
	1	6 (21%)	2 (7%)		16 (59%)	11 (37%)		9 (30%)	5 (17%)		3 (33%)	4 (29%)	
	2	12 (41%)	15 (50%)		4 (15%)	5 (17%)		0	3 (10%)		2 (22%)	2 (14%)	
	3	4 (14%)	11 (37%)		0	2 (7%)		0	0		1 (11%)	1 (7%)	
Steatosis													
Macro	<5%	0	30 (100%)		0	30 (100%)		0	30 (100%)		0	14 (100%)	
	5-35%	24 (83%)	0		24 (89%)	0		26 (87%)	0		8 (89%)	0	
	35-65%	4 (14%)	0		1 (4%)	0		3 (10%)	0		0	0	
	>65%	1 (3%)	0		2 (7%)	0		1 (3%)	0		1 (11%)	0	
Micro	<5%	1 (3%)	23 (77%)	*<0.001*	3 (11%)	24 (80%)	*<0.001*	2 (7%)	26 (87%)	*<0.001*	1 (11%)	9 (64%)	*0.14*
	5-35%	21 (72%)	5 (17%)		19 (70%)	5 (17%)		18 (60%)	4 (13%)		7 (78%)	5 (36%)	
	35-65%	6 (21%)	2 (7%)		3 (11%)	1 (3%)		9 (30%)	0		1 (11%)	0	
	>65%	1 (3%)	0		2 (7%)	0		1 (3%)	0		0	0	
Steatohepatitis													
	0	22 (76%)	29 (97%)	*0.03*	24 (89%)	30 (100%)	*0.09*	28 (93%)	30 (100%)	*0.20*	8 (89%)	14 (100%)	*0.40*
	1	6 (21%)	1 (3%)		2 (7%)	0		1 (3%)	0		0	0	
	2	0	0		1 (4%)	0		1 (3%)	0		1 (11%)	0	
	3	1 (3%)	0		0	0		0	0		0	0	

CK7+***						
0	7 (26%)	6 (21%)	*0.45*	11 (37%)	8 (36%)	*0.31*
1	6 (22%)	13 (56%)		6 (20%)	11 (50%)	
2	2 (7%)	4 (17%)		1 (3%)	3 (14%)	
3	0	0		0	0	

AIH, Autoimmune hepatitis; PBC, Primary biliary cirrhosis; PSC, Primary sclerosing cholangitis; MAFLD, Metabolic-associated fatty liver disease
Values given for N and (%).
*Including overlap disease with PBC or PSC
**Patients with stage 4 fibrosis in index biopsy were excluded
***Hepatocytes positive for cytokeratin 7, graded 0-3

Supplementary Table 5. Performance of NITs for detecting significant fibrosis (F2-F3). Cirrhosis (F4) excluded at study inclusion.

**FIB-4**	N	Prevalence*	sens	spec	PPV	NPV	AUROC	95% CI
AIH, MAFLD	27	40.7%	100.0/45.5%	18.8/31.3%	45.8/31.3%	100.0/45.5%	0.375	.158-.592
AIH, non FLD	181	36.5%	75.8/39.4%	25.2/53.0%	36.8/32.5%	64.4/60.4%	0.462	.376-.549
PBC, MAFLD	22	22.7%	60.0/20.0%	41.2/88.2%	23.1/33.3%	77.8/78.9%	0.553	.230-.876
PBC, non FLD	86	23.3%	90.0/30.0%	56.1/92.4%	38.3/54.5%	94.9/81.3%	0.772	.651-.893
PSC, MAFLD	27	18.5%	40.0/20.0%	68.2/100.0%	22.2/100.0%	83.3/84.6%	0.518	.136-.900
PSC, non FLD	151	23.8%	50.0/11.1%	77.4/94.8%	40.9/40.0%	83.2/77.3%	0.665	.558-.772
Toxic, MAFLD	8	25.0%	100.0/50.0%	16.7/66.7%	28.6/33.3%	100.0/80.0%	0.583	.192-.975
Toxic, non FLD	48	10.4%	80.0/80.0%	41.9/62.8%	13.8/20.0%	94.7/96.4%	0.702	.469-.936

**APRI**	N	Prevalence*	sens	spec	PPV	NPV	AUROC	95% CI
AIH, MAFLD	27	40.7%	90.9/36.4%	18.8/37.5%	43.5/28.6%	75.0/46.2%	0.437	.216-.659
AIH, non FLD	180	36.1%	87.7/44.6%	16.5/42.6%	37.3/30.5%	70.4/57.6%	0.420	.337-.503
PBC, MAFLD	22	22.7%	60.0%/n.c.	52.9/100.0%	27.3/n.c.%	81.8/n.c.%	0.529	.200-.858
PBC, non FLD	82	22.0%	83.3/22.2%	56.3/96.9%	34.9/66.7%	92.3/81.6%	0.793	.663-.923
PSC, MAFLD	27	18.5%	60.0%/n.c.	54.5/100.0%	23.1%/n.c.	85.7/81.5%	0.564	.210-.917
PSC, non FLD	151	23.8%	63.9/11.1%	60.0/94.8%	33.3/40.0%	84.1/77.3%	0.673	.580-.767
Toxic, MAFLD	8	25.0%	100.0/100.0%	16.7/50.0%	28.6/40.0%	100.0/100.0%	0.583	.192-.975
Toxic, non FLD	46	10.9%	100.0/80.0%	29.3/46.3%	14.7/15.4%	100.0/95.0%	0.620	.414-.825

**dAAR**	N	Prevalence*	sens	spec	PPV	NPV	AUROC	95% CI
AIH, MAFLD	27	40.7%	90.9/72.7%	25.0/37.5%	45.5/44.4%	80.0/66.7%	0.426	.206-.646
AIH, non FLD	183	36.6%	70.1/49.3%	26.7/38.8%	35.6/31.7%	60.8/57.0%	0.413	.332-.494
PBC, MAFLD	22	22.7%	80.0/20.0%	41.2/82.4%	28.6/25.0%	87.5/77.8%	0.612	.366-.858
PBC, non FLD	89	22.5%	90.0/55.0%	59.4/82.6%	39.1/47.8%	95.3/86.4%	0.807	.708-.905
PSC, MAFLD	28	21.4%	33.3%/n.c.	72.7/90.9%	25.0%/n.c.	80.0/76.9%	0.485	.216-.754
PSC, non FLD	152	23.7%	38.9/16.7%	77.6/88.8%	35.0/31.6%	80.4/77.4%	0.680	.588-.773
Toxic, MAFLD	8	25.0%	100.0/50.0%	16.7/50.0%	28.6/25.0%	100.0/75.0%	0.417	.025-.808
Toxic, non FLD	48	10.4%	80.0/60.0%	32.6/51.2%	12.1/12.5%	93.3/91.7%	0.684	.445-.922

Values given for lower/higher cut-off values. The cut-offs used: FIB-4: 1.3 and 2.67, APRI: 0.5 and 1.5, dAAR 1.79 and 2.63. N = number of patients in group with available NIT score.

*Prevalence of significant fibrosis (F2/F3) in patients with available NIT score.

Toxic = toxic liver disease

Sens; sensitivity, spec; specificity, PPV; positive predictive value, NPV; negative predictive value, MAFLD; metabolic-associated fatty liver disease, FLD; fatty liver disease, n.c.; no cases, NIT; non-invasive test, FIB-4; fibrosis-4-index, APRI; aspartate aminotransferase to platelet ratio index, dAAR; dynamic aspartate-to-alanine aminotransferase ratio

Supplementary Table 6. Performance of ultrasound for detection of biopsy-verified steatosis

	n	sens	spec	PPV	NPV	AUROC	95% CI
Macrovesic fat >5%	101	57.4%	87.2%	74.4%	76.0%	0.72	0.66-0.79
Macrovesic fat >35%	15	80.0%	72.7%	15.4%	98.3%	0.80	0.68-0.93
Microvesic fat >5%	126	44.4%	83.2%	71.8%	60.9%		
Microvesic fat >35%	29	58.6%	73.2%	21.8%	93.3%		

sens, sensitivity; spec, specificity; PPV, positive predictive value; NPV, negative predictive value; 95% CI, 95% confidence interval

Value for PPV in detecting macrovesicular fat >35% is accurate, low value is explained by vast majority of steatosis patients having <35% macrovesicular fat.
